# Macroscopic law of conservation revealed in the population dynamics of Toll-like receptor signaling

**DOI:** 10.1186/1478-811X-9-9

**Published:** 2011-04-20

**Authors:** Kumar Selvarajoo

**Affiliations:** 1 Institute for Advanced Biosciences, Keio University, Baba-Cho, 14-1, Tsuruoka, Yamagata, 997-0035 Japan

**Keywords:** cell signaling, governing law, systems biology, microscopic and macroscopic dynamics, immune response

## Abstract

Stimulating the receptors of a single cell generates stochastic intracellular signaling. The fluctuating response has been attributed to the low abundance of signaling molecules and the spatio-temporal effects of diffusion and crowding. At population level, however, cells are able to execute well-defined deterministic biological processes such as growth, division, differentiation and immune response. These data reflect biology as a system possessing microscopic and macroscopic dynamics. This commentary discusses the average population response of the Toll-like receptor (TLR) 3 and 4 signaling. Without requiring detailed experimental data, linear response equations together with the fundamental law of information conservation have been used to decipher novel network features such as unknown intermediates, processes and cross-talk mechanisms. For single cell response, however, such simplicity seems far from reality. Thus, as observed in any other complex systems, biology can be considered to possess order and disorder, inheriting a mixture of predictable population level and unpredictable single cell outcomes.

## Maintext

The innate immune cell, e.g. macrophage, upon recognition of external stimuli, such as a pathogen, invokes a sequence of molecular events, from receptor activation to gene expressions in the nucleus. This results in the induction of various proinflammatory cytokines that subsequently eliminate the intruders, usually through the adaptive immunity [1]. The well-orchestrated, self-organized and stable immune response, over a wide range and variety of perturbation, is observed at population level. However, recent reports at single cell resolution highlight the issue of cellular heterogeneity and stochasticity, switching attention to the variability and complexity of biological behaviors [2-4].

**Figure 1:**
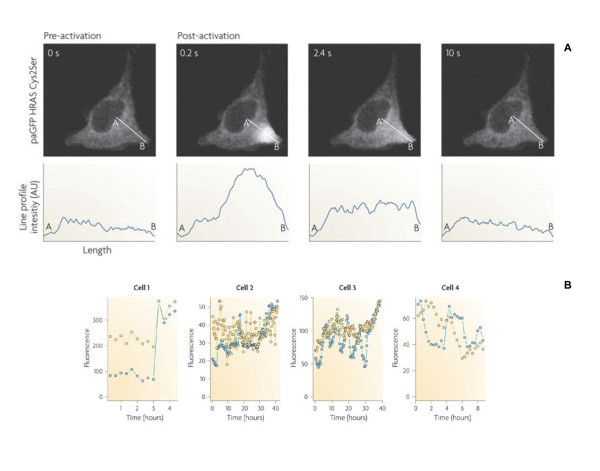
**Stochastic single cell behavior**. **A**) Illuminating green fluorescent protein (paGFP) with blue light on a single photoactivatable cell (upper panel) results in paGFP diffusing away from source in a stochastic manner, as shown by the intensity plots (lower panels). Intensity was measured in arbitrary units (AUs). **B**) Fluorescence levels for four individual cells show stochastic response. Blue circles represent the tumour suppressor protein p53 dynamics and the yellow circles represent the dynamics of ubiquitin E3 ligase MDM2. **A, B **adopted from [8] and [10], respectively.

A cell, within a population, possesses varying amounts of individual molecular constituents [4,5], in a highly inhomogeneous intracellular environment with spatio-temporal effects of molecular crowding and diffusion [6-9]. The low-abundance of numerous molecules produce stochastic cellular response or noise, such as in the dynamics of gene transcription and decay [2,10]. Together, the effect of space, crowding, stochasticity and heterogeneity of molecular constituents make single cell response variable, noisy and highly unpredictable (Figure 1A, B). On the other hand, cell populations display stable deterministic biological processes such as the synchronized collective dynamics of neuronal signaling. Hence, there is a need to distinguish the differences at the microscopic and macroscopic scales, so as to elucidate the causes for ordered response emerging from disordered response [11,12].

**Figure 2:**
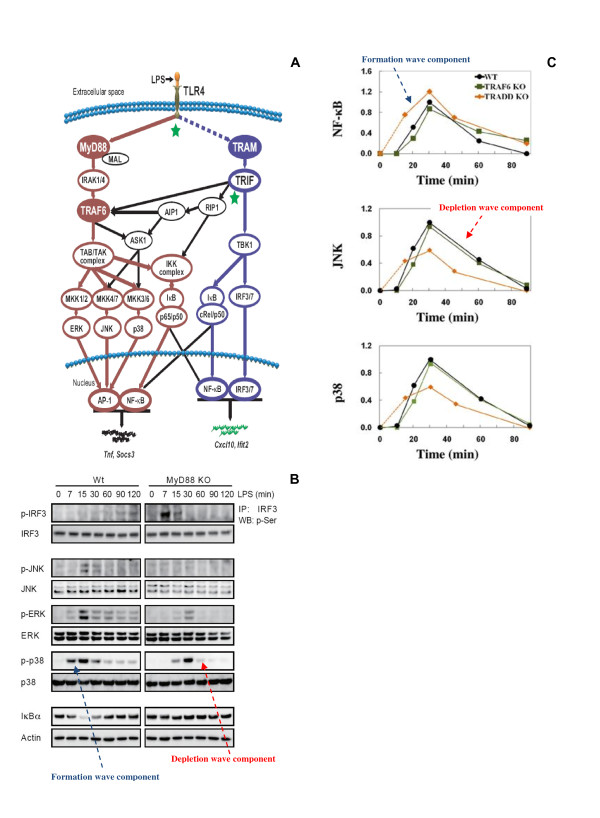
**Toll-like receptor signaling shows deterministic formation and depletion waves**. **A**) Schematic of TLR4 signaling. The dotted line between TLR4 and TRAM and the black lines indicate the prediction of novel intermediates [19] and crosstalk mechanisms [20], respectively. **B**) The western-blot activation profiles of IRF-3, JNK, p38 and NF-κB (degradation of IκBα) for LPS stimulation, and **C**) NF-κB, JNK and p38 profiles in wildtype or WT (black), TRAF6 KO (green), TRADD KO (orange) for poly (I:C) stimulation, show activation and deactivation following formation and depletion waves. **A, B **adopted from [18], and **C **from [21].

Over the past few years, our research has focused on the population level, well-characterized and co-ordinated dynamic signaling response of macrophages to invading pathogens based on the TLRs 3 and 4. Briefly, in TLR4 signaling, upon bacterial component lipopolysaccharide (LPS) recognition, MyD88 and TRAM molecules bind to TLR4 and trigger their respective pathways (Figure 2A) [1]. Notably, the experimental activation dynamics of immune-related proteins such as the NF-κB, JNK and p38, display response consisting of formation and depletion waves (Figure 2B, C). Instead of trying to measure each reaction's detailed kinetics, which faces huge technical challenges [8,13], we undertook a macroscopic view of developing a computational model based on perturbation-response approach and the law of information conservation.

**Figure 3:**
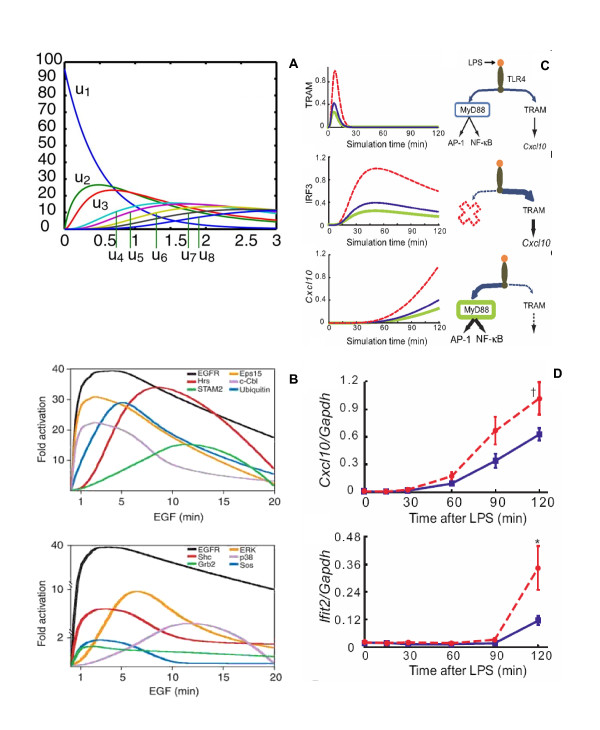
**Deterministic information conserved response of average cell signaling**. **A**) Temporal concentration response profiles of linear-chain reaction network for pulse perturbation of specie *X_1 _*(see maintext and [14]). The units are arbitrary, scaled by rate coefficients. *u1 *represents reaction profile for *X_1_*, *u2 *for *X_2 _*and so on. **B**) Temporal activation profiles of various EGFR signaling effectors: key proteins involved in receptor internalization and endosomal trafficking (upper panel) and proteins from the Ras-MAP kinase pathways (lower panel). **C**) The concept of *signaling flux redistribution *or *SFR*. Simulation profiles of proinflammatory molecules in wildtype (blue), MyD88 KO (red) and MyD88 overexpression (green). **D**) Experimental enhancement of TRAM-dependent molecules *Cxcl10 *and *Ifit2 *in MyD88 KO through *SFR*. Blue lines indicate WT and red dotted lines indicate MyD88 KO. **A **adopted from [14], **B **from [17] and **C, D **from [18].

The perturbation-response approach involves giving a small perturbation to the concentration of one or more reactant species in a network and analyzes the response profiles of other species within the network [14-16]. To briefly examine, consider a linear-chain of reactions (*X_1 _*→ *X_2 _*→ *X_3 _*→ ···) at steady-state condition. If the concentration of *X_1 _*is pulse perturbed, the concentrations of *X_2_*, *X_3_*, etc., will increase, go through a maximum, and then decrease back to its steady-state value in sequential order (Figure 3A). The experiments, based on the law of information conservation, connect the species between input and output fluxes through a linear superposition of propagation response waves (first-order response) [14-16]. Despite the simplicity of the approach, linear response is visually apparent in the dynamic phosphoproteomics data of several intracellular molecules activated by the perturbation of epidermal growth factor (EGF) receptors [17] (Figure 3B).

To illustrate further, a fixed perturbation of the input specie (e.g. LPS) generates downstream response waves of output species (e.g. p38 and JNK) that is conserved in terms of information propagation. The changes to an output specie's activations or concentrations, from steady-state or baseline levels, can be represented by the sum of formation and depletion terms:(1)

where *F_j_X_j _*and *F_k_X_i _*represent each formation (activation) and depletion (deactivation) term for the *i*^th ^molecule, respectively, and they can be any linear or non-linear function. Note that the number of formation and depletion terms, *p *and *q *for each *i*^th ^molecule, are variables obtained from the network topology of interest.

Given small perturbation to the generalized Eq.1, higher-order terms become negligible [15,16]. Hence, the partial differentiation in Eq. 1 can be changed into ordinary differentiation, i.e.  where ***X ***= (*X*_1_, *X*_2_,..., *X_n_*). Our previous works have used the approximation and have shown that a linear superposition of propagation response waves, or first-order mass-action response equations, can sufficiently be used to model the reaction chains of the TLR signaling [18-21]. This is valid especially for average cell response investigated for time points with a restricted range, usually before 120 min, where post-translational regulations (e.g. feedback or feedforward mechanisms) are insignificant [18,22].

Unlike typical kinetic models, which often use similar equations or sometimes with non-linear expressions to model the dynamics of biological networks, our perturbation-response approach considers the network as a sequence of events rather than molecules. As signaling networks are largely not fully understood, this difference is crucial as it prevents rigidly fixing the network topologies, and allows it to be modified according to experimental data so as to prevent overfitting problems and to identify novel features of signaling networks. In addition, as signaling process involves large number (thousands) of intracellular molecular activations, it is currently not plausible to model the dynamics of all possible reactions with the generally limited data. To overcome such difficulties, our approach permits the lumping of several molecules into a signaling specie in the model network. In this way, although the model does not become a comprehensive representation of an entire signaling process, however, it still allows the identification of overtly missing key features.

To successfully identify novel features of signaling networks, we set a target that the computational model should be able to simulate not just one experimental condition (like most models do), but in as many conditions as available. For the TLR4 signaling, we developed a wildtype model and compared the simulations with MyD88 and TRAF6 knock-outs (KOs), a total of three conditions. The initial wildtype model's parameter values for formation and depletion terms were determined directly by fitting simulations with quantified time-course activation experimental profiles of proinflammatory molecules (NF-κB, JNK, p38, *Tnf*, *Il6*, *Ifit1 *and *Cxcl10*) for LPS-stimulated murine macrophages [18,19].

Like any other modeling approach, there are certain limitations that require mentioning. Firstly, the perturbation-response approach discussed does not comprehensively represent the details of each signaling reaction's kinetics. Secondly, the small perturbation assumption leading to the first-order mass-action equations represents an average cell response and this cannot be used to study single cell stochastic behavior. Thirdly, the model predictions will show relative, and not absolute, activation levels. However, the approach is not restricted to the TLR pathways and can be applied to model any pathways that experimentally display formation and depletion waves, e.g. the EGF receptor signaling [17]. For information conservation to be observed, we need enough number of key output species to be monitored temporally.

In spite of the limitations, we predicted i) the presence of novel signaling intermediates along the TRAM-dependent pathways [19], ii) crosstalk mechanisms between the MyD88- and TRAM-dependent pathways [20], and iii) the concept of signaling flux redistribution or *SFR *[18]. The prediction of novel terms were later confirmed experimentally to be the phosphorylation of TRAM by PKCε and the sequential events of TLR4 endocytosis leading to TRAM activation [23,24]. The concept of *SFR *is based on the law of conservation where the removal of MyD88 resulted in the increased activation of the entire alternative TRAM-dependent pathway (Figure 3C, D). That is, the total signaling flux (information) propagation through the network from receptor activation through downstream gene activation is conserved. We experimentally validated *SFR *in two mutant conditions (MyD88 and TRAF6 KOs), where we observed increased activation of several alternative pathway molecules [18].

Similarly to the TLR4 signaling, we also investigated the TLR3 innate immune response against viral attacks by polyinosinic-polycytidylic acid (poly (I:C)). This leads to the activation of MAP kinases and NF-κB, which results in the induction of type I interferons and proinflammatory cytokines to combat the viral infection. Here, again analyzing the activation dynamics of the NF-κB, p38 and JNK in wildtype, TRAF6 KO and TRADD KO using a linear response model obeying the law of information conservation, we inferred i) the existence of missing intermediary steps between extracellular poly (I:C) stimulation and intracellular TLR3 binding, and ii) the presence of a novel pathway which is essential for JNK and p38, but not NF-κB, activation [21].

## Conclusions

Here I present, with examples from TLR 3 & 4 signaling in wildtype and several mutants, that the law of conservation and first order response equations are sufficient and important to reveal novel features of the complex immune process. This result is surprising as there is a general consensus that we need to fully understand all molecular interactions in the signaling network in order to make useful predictions [8,13]. Furthermore, the results from recent single cell experiments showing stochastic responses ask for spatial-temporal details to understand cellular signaling correctly [3,25].

So, instead of losing interest in the population level dynamics and move towards more single cell analyses, I propose biology is a system that possesses both microscopic and macroscopic dynamics, as observed in other physical sciences. For example, in the study of fluid dynamics, at microscopic level we observe the motion of each individual particle to be highly random and unpredictable and at macroscopic level, the velocity of airflow follows the fundamental law of fluid mechanics (the law of conservation of mass, energy and momentum). Thus, it is necessary to treat the two dynamics distinct.

It is also interesting to ponder the origins of averaging effect from stochastic response of a single cell when ensembles of them form a population. I believe that the emergence of average cell deterministic response from single cell stochastic response complement each other. For example, the stochastic fluctuations produced by a single cell are necessary to induce probabilistic differentiation from genetically identical cells [26-28]. This allows multi-cellular organisms to switch fates and states to yield diversity, such as for development or stress, which, otherwise, may be impossible from a purely deterministic system.

On the other hand, the well-coordinated response of cell populations, such as differentiation or growth, demonstrates that the single cell noise could cancel out when ensembles of cells are formed to generate a stable and robust response. Thus, the search for governing laws arising from single cell dynamics will enable us to better understand the coordinated response of cell populations. Most importantly, finding the connection between microscopic and macroscopic dynamics and the unifying laws are crucial for understanding the origins of evolutionary and developmental robustness of living systems to diverse environmental attacks.

## Abbreviations

MyD88: myeloid differentiation primary response gene (88); TRAM: TRIF-related adaptor molecule; TRIF: TIR-domain-containing adapter-inducing interferon-β; TIR: Toll/Interleukin-1 receptor; NF-κB; nuclear factor-κB; JNK: c-Jun N-terminal kinases; MAP; mitogen-activated protein; TRAF6: TNF receptor associated factor 6 protein; TNF: tumor necrosis factor; TRADD: Tumor necrosis factor receptor type 1-associated DEATH domain protein; IRF-3: interferon regulatory factor-3; IκBα: inhibitor of kappa B-α.

## Competing interests

The author declares that he has no competing interests.
